# Sample transformation in online separations: how chemical conversion advances analytical technology†

**DOI:** 10.1039/d3cc03599a

**Published:** 2023-11-15

**Authors:** Annika A. M. van der Zon, Joshka Verduin, Rick S. van den Hurk, Andrea F. G. Gargano, Bob W. J. Pirok

**Affiliations:** a University of Amsterdam, van’t Hoff Institute for Molecular Sciences, Analytical Chemistry Group Science Park 904 1098 XH Amsterdam The Netherlands B.W.J.Pirok@uva.nl; b Centre of Analytical Sciences Amsterdam Science Park 904 1098 XH Amsterdam The Netherlands; c Vrije Universiteit Amsterdam, Amsterdam Institute of Molecular and Life Sciences, Division of BioAnalytical Chemistry De Boelelaan 1085 1081 HV Amsterdam The Netherlands

## Abstract

While the advent of modern analytical technology has allowed scientists to determine the complexity of mixtures, it also spurred the demand to understand these sophisticated mixtures better. Chemical transformation can be used to provide insights into properties of complex samples such as degradation pathways or molecular heterogeneity that are otherwise unaccessible. In this article, we explore how sample transformation is exploited across different application fields to empower analytical methods. Transformation mechanisms include molecular-weight reduction, controlled degradation, and derivatization. Both offline and online transformation methods have been explored. The covered studies show that sample transformation facilitates faster reactions (*e.g.* several hours to minutes), reduces sample complexity, unlocks new sample dimensions (*e.g.* functional groups), provides correlations between multiple sample dimensions, and improves detectability. The article highlights the state-of-the-art and future prospects, focusing in particular on the characterization of protein and nucleic-acid therapeutics, nanoparticles, synthetic polymers, and small molecules.

## Abstract 2

Sample transformation can be used to provide insights into the properties of complex samples.

## Graphical abstract

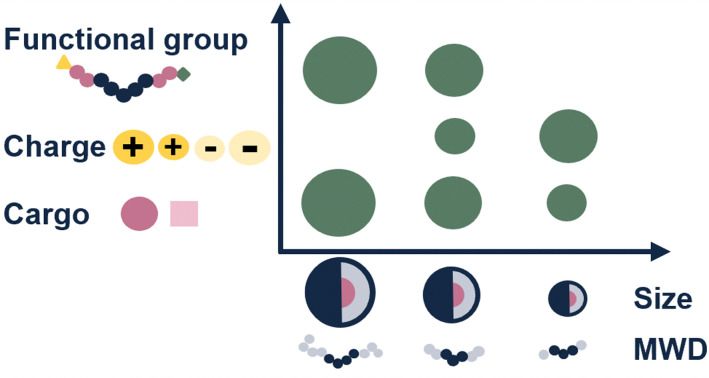

## 1. Introduction

The analysis of complex samples is a pervasive challenge in analytical chemistry. Progress in analytical science such as separation technology, mass spectrometry, and data analysis allow to identify and monitor hundreds of small molecules in complex matrices. The continuous progress in the area is allowing fields of research such as “omics” research to play an increasingly important role in various fields of biology, environment, clinical analysis, and more.^1,2^

Every chemical or technological innovation in a material, pharmaceutical formulation, or another product molecule is accompanied by analytical confirmation of its efficacy and safety. A case-in-point is material science, where increasingly sophisticated chemical strategies are being developed to create functional materials for, *e.g.*, the electronics industry (materials for chips, housing, coatings), high-tech manufacturing (materials for solar panels, 3D-printing), medicine (biomedical materials, drug excipients), and waterborne coatings. The applications of such polymers are also more and more specific. For example, some medical implants ideally last forever, whereas other ones are meant to disappear from the body at a given pace, without releasing any harmful degradation products. The latter is particularly relevant for the pharmaceutical industry, where drug-delivery systems are developed to deliver drugs to target locations in an organism. These systems must meet extremely strict regulations, and their composition must be determined in detail, including the carried amount of active pharmaceutical ingredient (API). Perhaps even more importantly, the stability of polymeric or pharmaceutical systems, the interaction between degradation products and API, and the effect of the degradation products on the patient, must be investigated. Similarly, modern pharmaceuticals such as monoclonal antibodies or additives to food must meet strict regulations, and their physical and product stability has to be verified.

### 1.1 Sample dimensionality

Each analytical method generally provides insight into a specific sample property. Defining a sample in key chemical properties (“sample dimension”) guides method development in chromatography.^3^ However, the samples subjected to analysis are increasingly complex and the analytical questions increasingly demanding, requiring accurate measurements of several of many sample dimensions and the correlation between these.

**Fig. 1:**
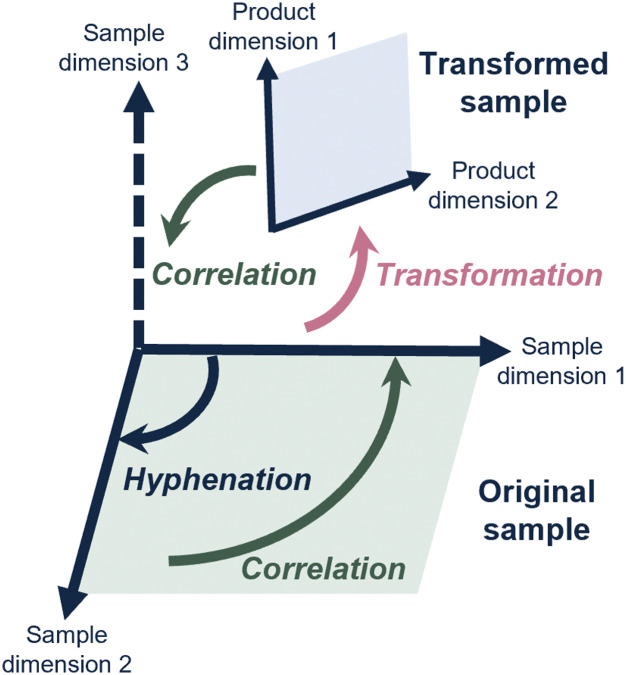
Schematic overview of how hyphenated analytical methods allow physicochemical sample properties (“sample dimensions”) to be correlated. By transforming the sample prior or during analysis, additional sample dimensions may be studied.

Consequently, scientists have hyphenated different analytical methods to simultaneously determine different sample dimensions. A well-known example, and workhorse in analytical chemistry, is liquid chromatography coupled with mass spectrometry (LC-MS). Most commonly, this concerns reversed-phase LC (RPLC) to assess the hydrophobicity, combined with, and correlated to, a determination of mass-over-charge through the MS. This concept is depicted in Fig. 1, where hyphenation (dark blue arrow) allows the determination of a second sample dimension, which can then also be correlated to (green arrow) the first sample dimension.

Similarly, chromatographers proceeded to combine two LC separations in comprehensive two-dimensional liquid chromatography (LC × LC). Valuable information is obtained when fractions from a first LC dimension are studied with a completely different (“orthogonal”) LC method, to obtain a second, completely independent, characterization and to drastically increase the separation power of the system.^4^ Consequently, LC × LC has successfully been applied to study complex samples for different applications in industry over the last eight years.^5^

However, for many relevant challenges described above, the analytical question involves a sample dimension for which no analytical method exists, or that is difficult to achieve by simply hyphenating analytical methods (depicted in Fig. 1 as “sample dimension 3”).

### 1.2 Sample transformation to study sample properties

An interesting solution to this problem is the use of reactors to change the sample prior to or during analysis methods. The advantage of this can be pictured in different ways.

One advantage is that it theoretically allows the correlation between the property of the sample in native conditions and another property of the altered sample. For example, proteins are currently studied either at intact level, and middle-up or a bottom-up method where the protein is digested and the fragments are analyzed. Sample transformation within an analytical workflow could allow the characterization of an intact protein (sample dimension 1), and – after being subjected to a reactor, its fragments (sample dimension 2) could be studied as a function of the intact protein, all within one experiment. For a drug-delivery system (DDS) this could be imagined as the study of intact DDS particles, a controlled deconstruction of the particle, and analysis of the contents within one experiment. Another advantage is the ability to study degradation mechanisms. Here, the conditions that may change the molecular properties of a sample over time may be investigated.

Sample transformation can be implemented in analytical workflows either separately from the analysis (*i.e.* offline) or coupled directly with the analysis method (*i.e.* online). Online methods typically allow for faster reactions (within minutes), reducing sample handling steps. Examples include the use of immobilized-enzyme reactors (IMERs), electrochemical cells, solvent mixing units, pyrolysis, and photochemical reaction cells. The evolution of systems performing online transformation has recently sparked development towards the implementation of transformations in analytical platforms such as multi-dimensional liquid chromatography (mD-LC) workflows. This approach, referred to as online sample transformation, allows measuring and resolving complex sample components and applying the transformation to the discrete portion of the initial sample.

**Fig. 2:**
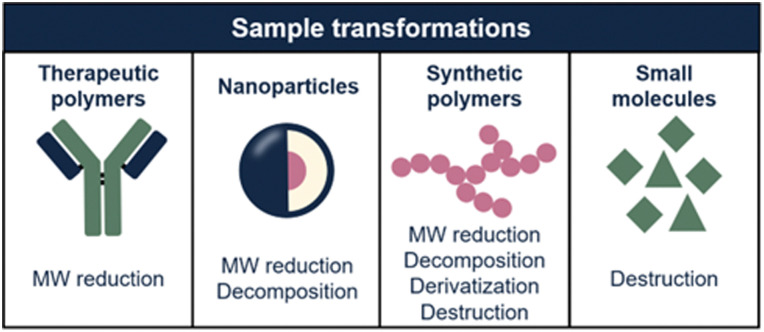
Overview of the sample transformations that are applied per sample type (*i.e.* therapeutic polymers, nanoparticles, synthetic polymers, and small molecules). Implementation of sample transformation unlocks the ability of analyzing multiple sample properties.

In this article, we explore how sample transformation is exploited across different application fields to empower analytical methods. The studied sample dimensions and, consequently, transformation mechanisms, differ highly for each application field (Fig. 2). This review is structured accordingly, focusing on protein and nucleic-acid therapeutics, polymeric nanoparticles (NPs), synthetic polymers, and small molecules. Each chapter comprises of background information regarding the discussed sample class, challenges in characterization, and how sample transformation methods can be incorporated into separation-based analytical workflows. State-of-the-art results and future perspectives of this research area are discussed.

## 2. Protein and nucleic-acid therapeutics

### 2.1 Introduction

Within biotherapeutic polymers, have enabled significant progress in the treatment of diseases, such as inflammatory, autoimmune disorders, infection, oncologic, and cardiovascular diseases. Examples of emerging biotherapeutics include polysaccharides (*e.g.* fungal polysaccharides), protein and nucleic acid therapeutics.^6,7^ However, polysaccharides are not mentioned in this review due to the different heterogeneity in contrast to a natural polymer. In this section, the latter two therapeutics and how sample transformation is used to characterize their structures is covered. In the last seven years, more than 125 drugs of this type have been approved by the US Food and Drug Administration (FDA).^8^ Examples of protein-based therapeutics include monoclonal antibodies (mAbs), antibody–drug conjugates (ADCs), and Fc-fusion proteins. mAbs are proteins designed to bind to a specific target, such as a protein on the surface of a cancer cell, and trigger immune responses to eliminate or neutralize the target and treat the disease.^9,10^ ADCs are mAbs that are conjugated to a drug payload (mostly cytotoxic molecules) via a chemical linker. ADCs are an upcoming therapeutic used for oncology.^11,12^ Lastly, Fc-fusion proteins combine the Fc region of an antibody with a therapeutic protein, enhancing its stability and half-life, and allowing it to selectively target specific receptors or molecules, thereby improving its efficacy and therapeutic potential.^13,14^

Nucleic-acid therapeutics are a growing class in human therapeutics altering the expression of deoxyribonucleic acid (DNA) or ribonucleic acid (RNA) for therapeutic purposes.^15^ There are a variety of DNA and RNA-based therapeutics, including antisense oligonucleotides (ASOs), microRNAs (miRNAs), small interfering RNAs (siRNAs), and aptamers. Oligonucleotides (ONs) are all short, single (ASOs, miRNAs) or double (siRNAs) stranded polymers composed of synthetic nucleic acids.^16,17^

### 2.2 Characterizing protein and nucleic-acid therapeutics

The high molecular weight (MW) and heterogeneity of protein and nucleic-acid therapeutics limit the information that can be obtained when studying them as intact. Therefore, sample transformation workflows are essential to identify and characterize critical quality attributes (CQAs).^18^ Characterization of protein-based (mAbs, ADCs, fusion proteins) and nucleic-acid-based (ONs, RNA) therapeutics is challenging. These biopolymers can have various degrees of heterogeneity such as functional chemical modifications or higher-order organization that must be monitored. Proteins could undergo enzymatic and chemical modifications, known as post-translational modifications (PTMs).^19^ Similarly, nucleic acid can have post-transcriptional modifications. Common modifications that can occur in protein- and nucleic-acid-based products are sequence variants, deamidation, oxidation, and chemical modifications.^20,21^ In addition, protein-based and some nucleic-acid pharmaceuticals, such as ONs, can have polysaccharides (glycans) attached to specific amino acids. This is referred to as glycosylation. These modifications can affect the activity of pharmaceuticals, for example in mAbs glycosylation and for ADCs, the drug-antibody-ratio (DAR) is important to determine the efficacy of the drug.^22^

For RNA therapeutics, chemical modification such as backbone modifications, sugar modifications, base modifications, and conjugates is key to improving the pharmacokinetic behavior of the ONs.^23^ Because of the influence of PTMs on the safety and efficacy of biotherapeutic products, it is critical to understand the type and localization of the specific modification. Advanced analytical methods are required to provide a better characterization of protein structure and identify modifications. LC-MS is the most common approach applied for such characterization. However, due to the complexity and large size of these molecules, the characterization of biopolymers at the intact level is challenging. The size of the PTMs is minor (from 1 Da to ∼300 Da) in contrast to the high molecular weight of the biopolymer (generally over in the tens to hundreds of kDa). As a result, separation and mass spectrometric methods are limited in the degree of information they can provide on the presence and localization of chemical or structural alteration of the heterogeneity of biopolymers. Therefore, to obtain a detailed characterization of pharmaceutical biopolymers, multilevel analytical strategies allow the study of specific portions of the large molecules. These analytical workflows rely on sample transformation approaches that selectively reduce the molecular weight and/or sample complexity by altering or removing post-translational or transcriptional modification. In our discussion, we classify methods as non-enzymatic and enzymatic.

### 2.3 Sample transformation for therapeutic biopolymers

#### 2.3.1 Non-enzymatic transformation

We first focus on the non-enzymatic transformation of chemical and electrochemical reactions used to perform biopolymer cleavage and subsequent characterization. By means of these, cleavage of protein or nucleic acid happens in the presence of specific amino or nucleic acids or of specific bonds.^24^

To study protein-based therapeutics, weak organic acids such as formic acid and acetic acid can be used, cleaving preferentially aspartic acid residues. Strong inorganic acids (*e.g.* hydrochloric acid) are less selective than weak organic acids. Other chemicals used for chemical cleavage include (i) cyanogen bromide which cleaves at the C-terminal side of methionine residues, (ii) 2-nitro-5-thicyanobenzoate which cleaves on N-terminal cysteine residues, and (iii) hydroxylamine which hydrolyzes asparagine and glycine.^25–28^ Reduction reactions are applied in particular to break the crosslinks between protein portions or sub-units mediated by bonds between the sulfide groups of cysteine amino acids. Reagents such as tris(2-carboxyethyl) phosphine hydrochloride (TCEP), dithiothreitol (DTT), or mercaptoethanol are used, reducing disulfides to free dithiol. Often, these are used in combination with denaturing agents such as urea and guanidine hydrochloride, to unfold the protein structures. In addition, electrochemical oxidation can be used to reduce disulfide bonds^29,30^ and/or specifically break peptide bonds.^31^ Chemical and electrochemical reactions to reduce the MW of the protein polymers are often quick and can be performed within minutes.^32^

Basile and Hauser in 2011 demonstrated the online coupling of chemical hydrolysis and electrochemical oxidation for the reduction of reference proteins (MW 3–67 kDa), and a cell lysate (*Escherichia coli*).^32^ In their study, they implemented online microwave heating acid (formic acid (FA)) to hydrolyze proteins at aspartic acid residues combined with online electrochemical oxidation at tryptophan and tyrosine residues. A typical limitation of electrochemical oxidation is that it yields large peptides, with respect to enzymatic digestion. However, Basile and Hauser showed that with their setup the generated peptide length was similar to peptides that were digested *via* an enzymatic way (nine amino acids). The total digestion time of the proteins was six minutes. The authors claimed that incorporating this approach in a multi-dimensional system can increase the efficiency of the combined non-enzymatic digestion and the number of detected proteins. Recently, Morgan *et al.* implemented an online electrochemical reduction to perform RPLC-MS analysis of a reference (NIST) mAb at the middle-up level.^33^ The authors showed that with an electrode flow-through cell equipped with a platinum counter electrode, the antibody can be reduced under certain electrochemical potentials, temperature, and organic solvents. A complete reduction of the heavy and light chains was performed without prior sample preparation. The antibody was eluted in five minutes from the electrochemical cell into a trap column and analyzed with RPLC-MS. The simplicity of online hyphenation of the electrochemical cell reactor renders this approach attractive in multi-dimensional separation systems. The antibody could then be analyzed at intact and middle-up levels to obtain more information about the PTMs.

Electrochemical approaches are not commonly applied online. This could be because of their complexity and need for specific devices. In contrast, setups to perform chemical modification are less sophisticated. For example, reduction and denaturation of proteins can be implemented in online LC-MS workflows in combination with enzymatic digestion. For antibodies, such reduction was successfully performed using an RPLC analytical column to retain the protein and using DTT or TCEP in the mobile phase to reduce the protein. The reduction takes place in minutes and can be performed at room temperature or to speed up the process under heating (*e.g.* up to 80 °C).^34–38^

Chemical hydrolysis approaches for nucleic-acid-based therapeutics have been investigated over the last decades. With acidic hydrolysis, it is possible to reduce the DNA size or RNA size to small repeating units. Lowenthal *et al.* demonstrated an acidic hydrolysis approach for intact DNA and for the first time applied to ribonucleotide oligomers hydrolysis.^39^ The authors tested three types of acid approaches; (i) trifluoroacetic acid (TFA), degradation of nucleobases within an hour tested at 60–140 °C, (ii) hydrochloric acid degradation of nucleobase pyrimidines and purines within 1–4 hours and (iii) FA degradation of pyrimidines and cytosine for 24 hours at 140 °C. With a prolongation time (24–48 hours), the nucleobases hydrolyzed completely from intact DNA and RNA.^40^

#### 2.3.2 Enzymatic digestion in solutions

Enzymatic reactions are often used for structural characterization given their high selectivity towards chemical groups or biopolymer sequence motifs. Workflows using enzymes are often referred to as middle-up or bottom-up analysis for protein characterization (bottom-up generally below 3 kDa, middle-up above 10 kDa) and enzymatic sequence mapping. Depending on the selectivity of the enzyme, amino acid sequences and/or PTMs can be studied.

In protein pharmaceutical analysis, bottom-up proteomics can be applied for the identification of the amino acid sequence and variations in it as well as to characterize the location of a certain PTM in the protein sequence. Trypsin is the most widely used protease because of its high cleavage specificity.^25,41^ The enzyme hydrolyzes the protein at C-terminal lysine and arginine amino acids into peptide fragments. In a typical offline trypsin in-solution digestion profile, the protein will be denatured, reduced, and alkylated before digestion. The total in-solution digestion time depends on the application, but it may take up to 18 hours.

Alternative enzymes for trypsin are endoproteinases such as Lys-C (cleaves lysine residues), pepsin (cleaves leucine, tryptophan, tyrosine, and phenylalanine), and papain (cleaves arginine, lysine, aspartic acid, histidine, glycine, and tyrosine) which have a high cleavage efficiency and target other amino acids. Switzar *et al.* listed commonly used enzymes for protein digestion. For studying the proteins more in detail, multiple proteases are used to allow to cover the protein sequence with cleavages performed at different sites.^25^

Middle-up approaches can be used to generate fragments with molecular weight generally between 20 and 50 kDa. This allows for retaining information on a large portion of the protein sequence and studying the co-occurrence of modifications.

For mAbs, ADCs, and fusion proteins, the immunoglobin-degrading enzyme IdeS from *Streptococcus pyogenes* is often used. This enzyme is a cysteine proteinase that cleaves at the Gly236–Gly237 at the hinge region of immunoglobin G (IgG) to generate F(ab′)2 and Fc/2 fragments. In middle-up approaches, the digestion time is often shorter than in bottom-up approaches being generally between 30 to 60 minutes.^42^

**Fig. 3:**
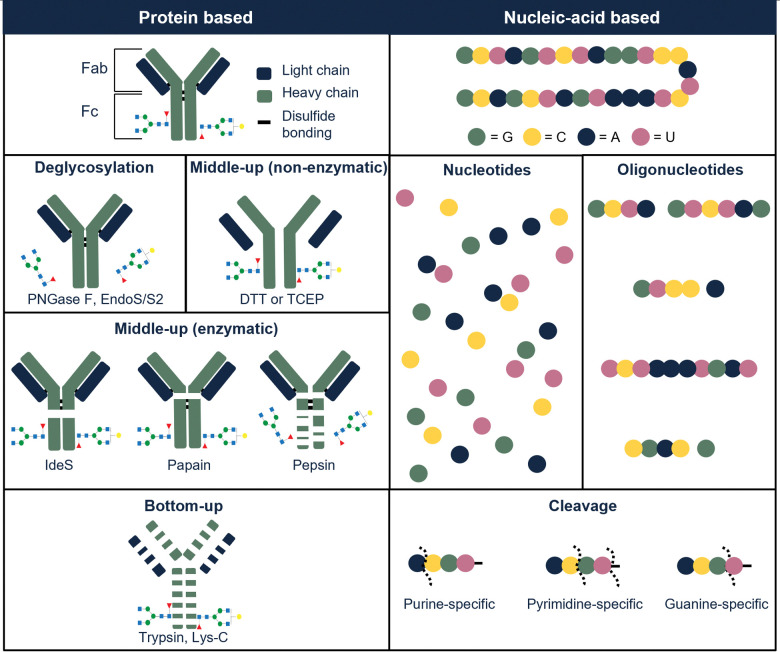
Illustration of commonly used digestion approaches used to characterize protein-based therapeutics (left part of the table), illustrated with examples from protein-based therapeutics (illustrated with example of mAb) and nucleic-acid-based therapeutics (illustrated with example from RNA-based molecule).

Glycosylation is a common PTM in therapeutic proteins, where a polysaccharide (glycan) is covalently bonded to a protein. Glycans typically have chemical composition and molecular weight distributions, increasing the heterogeneity of the drug. When characterizing glycoproteins, both protein, and polysaccharide(s) have to be studied. Several glycoside hydrolases are present, enabling to hydrolyze specific bonds or glycans in the presence of specific sugars. An enzyme that is commonly used is *N*-glycosidase F (PNGase F) which effectively removes *N*-glycans from glycoproteins by cleaving between the *N*-acetylglucosamine and asparagine residues.^43^ This enzyme can be used on the intact or on the hydrolyzed therapeutic. Fig. 3 illustrates the most commonly used enzymes for protein therapeutic characterization using mAbs as an example. Molina and Camperi listed other enzymes that can be used in mAb-based characterization.^44^ Commercial liquid handling or automatic purification systems can be used to perform proteolytic digestion. An example is the automated in-solution digestion with a common HPLC system developed by Richardson *et al.*^45^ However, the long digestion times that are frequently needed for in-solution enzymatic reactions such as trypsin digestion, can be a risk for inducing artificial modifications such as oxidation and deamidation.

Another way of performing online sample transformation in solutions is to use in-capillary or in-loop digestions. Results have shown that compared to traditional offline methodologies in-capillary strategies, thanks to advanced mixing, can reduce both the total analysis time (*e.g.* between 15 min to 4 h) and the volume of reactants by a factor of 4 and 1000 respectively.^46^ This approach has been demonstrated for most of the offline enzymatic approaches and coupled to LC as well as capillary zone electrophoresis (CZE).^46–49^

The reduced sample requirements, automation, and reduced reaction times have allowed performing online sample transformation, using in-capillary digestions in multi-dimensional separations. An example is the work of Mayr *et al.* where an automated in-capillary digestion was used to perform various enzymatic reactions within a heart-cut multi-dimensional system.^50^ Up to 6 fractions from an ion exchange chromatography (IEC) charge variant separation of monoclonal antibodies were collected and in-capillary digestion allowed for fragment generation (bottom or middle-up), or deglycosylation followed by RPLC-MS. The digestion times varied between minutes and hours depending on the reaction performed.

Recently, Schlecht *et al.* showed a two-dimensional CZE-MS application with in-capillary reduction and digestion by using pepsin and TCEP in a fused-silica capillary.^51^ In the first dimension, the intact mAb was separated based on charge variants followed by in-capillary digestion and separation of selected charge variants. The digestion time was ten minutes. They concluded that other enzymes for peptide mapping or subunit analysis can be used in their setup after further optimization. The total digestion time was around ten minutes.

In-solution enzymatic digestions are also widely used for nucleic-acid therapeutics characterization. Several types of nucleobase-specific (nucleases and phosphatase) or sequence-specific ribonuclease (RNase) can be used to digest RNA into nucleosides and ONs and reduce the molecular weight of nucleic-acid-based therapeutics. Both types of enzymes can help in order to identify sequence variants and post/co-transcriptional modifications, and nucleobase modifications. Expected digestion products of RNA sequences are listed in the publication of Thakur *et al.*^52^ Recently, Fekete *et al.* reviewed several digestion strategies for RNA and ONs.^53^ The most commonly used RNases are RNase T1 which cleaves at guanosine and RNase A which cleaves at pyrimidines.^54^ Uridine-specific ribonuclease enzymes such as RNase 4 and MC1 cleavage at the uridine gives additional sequence information. RNase U2 is a purine-specific enzyme. RNase T1, RNase A, and RNase U2 are commercially available. Other enzymes that can be applied are (i) cusativin, a cytidine-specific enzyme, (ii) colicin which cleaves between glycine and selenocysteine, (iii) Maz F which cleaves at alanine–cysteine–alanine are produced in-house. Fekete *et al.* concluded that longer digestion protocols are required to obtain enhanced sequence coverage compared to RNase T1 with these enzymes.^53^ As shown, there are multiple different enzymes possible for nucleic-acid-based applications. Combining various enzymes can result in a higher sequence coverage and more information on RNA can be obtained. Jiang *et al.* developed parallel RNase digestion for ONs with LC-MS/MS, whereas Goyon *et al.* used parallel RNase digestion for full sequencing of single-guide RNA with LC-tandem MS (MS/MS)^55,56^ The enzymes (RNase T1, colicin E5, and MazF) that were used in the study of Jiang *et al.* reached a sequence coverage greater than 70% with long peptides (near 3000 nucleotides).^55^ They concluded that the RNase T1 generates shorter ON digestion products compared to colicin E5 and MazF due to their specificity. The total digestion times of these RNase enzymes take between 15–30 minutes depending on the type of enzyme. However, in both studies, the digestion methods were performed in an offline way which is laborious and time-consuming. Additionally, RNase digestion products can result in RNase contamination which can harm the LC-MS performance.^57^

#### 2.3.3 Enzymatic digestions using immobilized-enzyme reactors

Performing enzymatic digestions online can (i) save significant operation time, (ii) reduce the generation of sample handling-related modifications (*e.g.* oxidation and deamidation), and (iii) reduce the contamination with respect to the in-solution approaches.^44^ For performing online enzymatic digestions, flow-through IMERs have been developed.

IMERs are cartridges that contain an enzyme covalently immobilized on a solid surface (in most cases, polymer-packed beds).^58^ Compared to in-solution methods, the immobilization of enzymes allows for an increase of enzyme/substrate ratio, resulting in shorter digestion times (minutes or seconds) accurate (low number of miss cleavages; sequence coverages above 98%) digestions, and reduced enzyme autolysis.^59,60^

In the last five years, numerous papers have been published about the sample transformation of mAb using IMERs both as an online combination to LC-MS as well as in a multi-dimensional system. Several commercial products based on IMERs are available and the enzyme types are limited to trypsin, Endoproteinase LysC, and IdeS.^59^ Table S1 of the ESI,† shows an overview of the IMERs used for therapeutic biopolymers in online platforms. The robustness and reproducibility of multi-dimensional separation including IMERs online sample transformation were demonstrated by an interlaboratory study of Camperi *et al.* in 2021.^36^

**Fig. 4:**
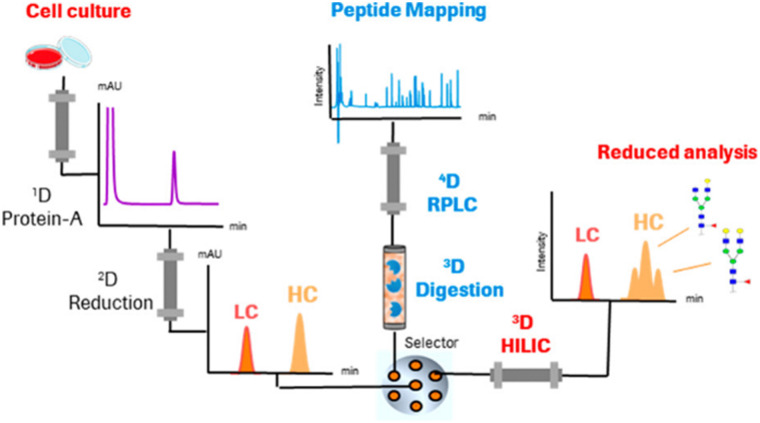
Schematic representation of a multi-dimensional system that includes online reduction and digestion of monoclonal antibodies. Reprinted (adapted) with permission from *Anal. Chem.* 2020, 92, 12, 8506–8513. Copyright 2020 American Chemical Society.^35^

Studies applying online IMERs often describe sample transformation as a separate dimension in the characterization workflow (see Table S1, ESI†). In the three-dimensional LC-MS systems listed in Table S1 (ESI†), the three dimensions consist of 1D: reduction – separation by RPLC, 2D: digestion – IMER, and 3D: peptide mapping – RPLC. One of the first examples of a fully automated system mD-LC-MS was published for the characterization of antibody variants by Gstöttner *et al.* in early 2018. They developed a 1.5 hour run in which the variants are separated by IEC coupled to online reduction and trypsin digestion. This allowed characterization of peptide modifications, such as succinimide, isomerization, and deamidation.^37^ However, this run time is for collecting one peak in the IEX run. The peak was trapped and reduced on an RP column. Then, in the third dimension, online trypsin digestion was performed where the digest products were separated in the last dimension. Improvements to this mD-LC-MS setup have been reported over the years in which ultra-fast digestion within one to two minutes was achieved, see Table S1 (ESI†). Fig. 4 shows a setup of Camperi *et al.* where they combine reduced analysis and peptide mapping in one setup.^35^

Next to trypsin digestion, other enzymes have also been used for online digestion using IMERs. Camperi *et al.* used IdeS in the first dimension to reduce the complexity of the antibody.^61^ Whereas, Oezipek *et al.* used an in-parallel IMER with LysC and trypsin digestion in their mD-LC-MS setup.^62^

Only a few reports describe the use of IMERs for the characterization of ADCs. In 2019, Goyon and co-workers described a mD-LC-MS (SEC-reduction-digestion-RPLC-MS) setup with an included IMER sample transformation step.^63^ In their setup, SEC was used to separate size species under non-denaturing conditions. To determine aggregates at a middle-up level, DAR, and PTMs at the peptide level, a reduction, and digestion (trypsin IMER) step was implemented with an RPLC separation in the mD-LC setup. IMERs have also been used for the analysis of fusion proteins. Recently, the group of Camperi developed an mD-LC-MS platform with online reduction and digestion (trypsin or Lys-C) for peptide mapping of Fc-fusion proteins.^64^ Multiple attributes such as oxidation, deamidation, proline amidation, N-terminal glutamine cyclization, C-terminal lysine clipping, glycosylation, and succinimide formation were studied next to the characterization of multiple *N*-glycosylation sites. A follow-up by Camperi's group applied this for real-time monitoring of attributes during the production process of cell cultures.^65^

IMERs can also be implemented for nucleic-acid-based therapeutics. However, in contrast to protein-based systems, only prototype based on a few RNase enzymes (RNase T1, A, and U) are available.^66^ Butterer *et al.* demonstrated that the use of RNase IMERs is advantageous in the analysis of nucleic-acid therapeutics as it limits the RNAse contamination in the digested sample.^57^ In their study, they used a commercial RNase A and custom-made immobilized RNase T1, RNase A, and RNase U2 IMERs. The digestion was performed offline and their time was between 30–120 minutes depending on the enzyme. Goyon *et al.* published, for the first time an automated digestion using a prototype ribonuclease IMER cartridges for sequencing of CRISPR guide RNAs in an mD-LC-MS/MS platform.^66^ RNase T1, A and U2 were immobilized in polyetheretherketone cartridges. The IMERs were coupled in parallel before hydrophilic interaction chromatography (HILIC)-MS. Depending on the approach of interest, the valve could be switched to a specific IMER. Using these IMERS, the digestion time was 2.3 minutes compared to traditional digestion of 15–30 minutes. According to the authors, the highest sequence coverage (71%) was obtained with the RNase T1 IMER. By combining the data of all three IMERs, a sequence coverage of 84% was obtained. With the use of these IMERs, the sample amount was significantly reduced. In their follow-up study, impurities of large therapeutics RNAs at the nucleotide level were identified by using the RNase T1 IMER.^67^ The digestion time was here under three minutes. These two studies showed that there are opportunities for faster enzymatic digestion and minimizing the introduction of modifications.

## 3. Nanoparticles

### 3.1 Introduction

Nanoparticles are macromolecular systems with a size in the nanometer range.^68^ They are widely used in cosmetics, materials, and pharmaceuticals. They are also released into the environment as a result of human activity and therefore studied in environmental sciences. They can be composed of various materials, including metals, metal oxides, semiconductors, carbon-based materials, and polymers. Depending on the application field, different size characteristics are attributed to nanoparticles. In environmental sciences, NPs are commonly polymer based and therefore referred to as plastics with a size range up to 100 nm, whilst submicron particles are sometimes referred to as microplastics.^69^

NPs are complex systems, often formed by multiple components, and of large size. Their analysis often requires investigating multiple attributes (*e.g.* size distribution and constituents) and is complex. For the purpose of this review, we will focus exclusively on sub-micron polymeric NPs and adeno-associated viruses (AAVs).

### 3.2 Characterizing nanoparticles

The characterization of polymeric NP comprises the determination of the (i) particle-size distribution (PSD) and identification of (ii) the NP components (*e.g.* carrier, surfactants, encapsulated compounds, polymer components, and their ratios). PSD assessment is important for all NPs as it strongly influences NP properties and is therefore an important parameter for production and safety risk assessments. During fabrication, it is typically desired to obtain NP samples with a monodisperse size distribution, making PSD determination important for quality control.^70^ PSD analysis is also important to determine the uptake of NPs in the environment or organisms, which can either be wanted or unwanted depending on the application. In the environmental field, pollution by NPs in nature and living organisms is of concern.^71^ As an example, Leslie *et al.* recently demonstrated the presence of plastic NPs in the human body.^72^ For medicinal NPs, however, administration of the nanomedicine is key. These DDSs are nanoparticles that have an API encapsulated. A well-known example is the lipid NP vaccines that have been developed during the COVID-19 pandemic.^73^ For DDSs, PSD determination is important as particle size and shape determine the uptake, encapsulation efficiency, and release of the pharmaceutical.^74,75^

The complexity of the analysis of the NP constituents depends on the type of NP. NPs can either consist of a single component (*i.e.* one polymer) or multiple components. The simplest polymeric NP is one that only consists of a single polymer, such as polystyrene. The polymer characterization itself is challenging, as it requires the assessment of multiple properties (*e.g.* repeating unit, molecular weight distribution, end group characterization). This will be discussed in more detail in Section 4. However, most NPs have a more complex composition. For instance, surfactants are often added to the sample matrix to stabilize the formulation or enhance its functionality.^76,77^ In addition to surface modification, NPs could also contain an encapsulated compound. For instance, APIs or additives in medicinal and food NPs, respectively.^77^ This leads to additional characterization of the encapsulated compound but often also requires quantification of the cargo.

All these different NP attributes are often analyzed with separate analytical methods.^78–80^ Potentially, the implementation of a sample transformation step to facilitate the assessment of multiple sample dimensions at once could be advantageous. This would not only improve the method's efficiency but also allows for obtaining the correlation between different sample attributes. The implementation would also make the analysis less laborious and therefore more time efficient.

### 3.3 Sample transformation for polymeric nanoparticles

#### 3.3.1 Thermal transformation

In the case of environmental nanoparticles, particle size is often the main property of interest, and therefore no large variety of sample transformations has been described in literature Table S1 (ESI†). However, thermal transformation is commonly reported to assess environmental micro- and nanoplastics, with pyrolysis gas chromatography (Py-GC) being a useful hyphenated technique.^69,72,79–81^ In the field of polymers, Py-GC-MS is one of the most established tools to study the monomer composition. Readers interested in Py-GC-MS for this application are referred elsewhere.^82^ During pyrolysis compounds are rapidly heated during which weak bonds are broken.^80^ Consequently, the samples are decomposed into smaller fragments. The subsequent GC-MS analysis allows for the identification of the polymer (*e.g.* type, sequence distribution), additives, and in some cases quantitation.^83^

#### 3.3.2 Enzymatic digestions

Despite not being as established as biopolymers, a few studies exist that have reported on the enzymatic transformation of polymeric NPs. In the case of environmental NPs, not only the polymer itself, but the sample matrix could be too complex to analyze directly. These environmental samples commonly need to be purified to extract the NP from the sample matrix. For instance, Rai *et al.* made an overview of the standardization of sample preparation methods for environmental micro- and nanoplastics.^84^ One of the described methods to purify the samples included the use of enzymes (*e.g.* microbial, proteolytic, cellulase), oxidants, or a combination of these. However, these methods were not performed online.

Enzymatic digestions can also be applied to medicinal NPs. These can be coated with a layer of proteins, also referred to as protein coronas. To study protein binding and quantification, the removal of the proteins from medicinal nanoparticles with trypsin has been demonstrated.^85,86^

Whilst already studied and implemented in the field of biopolymers, the application of IMERs on synthetic polymers is virtually unexplored. In 2019, we attempted to perform online enzymatic digestion on polyester NPs using an IMER that was prepared in-house.^87^ The co-polymer used consisted of two outer blocks of 6–12 kDa and a middle block of 4 kDa. After a comparison of in-solution digestions of the polyester with lipase and trypsin, it was found that lipase had the fastest degradation time. Therefore, lipase was implemented in the IMER to perform online digestions with a residence time of two minutes. The IMER effluent was analyzed online with SEC-ELSD and the results indicated degradation of the polymer. Yet, identification of the formed degradation products was not performed. Nonetheless, this study demonstrated the proof-of-principle of using IMERs for the degradation of synthetic polymers.

Besides polymeric NPs, enzymatic approaches have also been reported for the analysis of AAVs. AAVs are a platform for gene delivery that is being studied for the treatment of a variety of human diseases.^88^ AAVs are composed of a protein capsid shell formed by several repeating proteins and contain DNA or RNA. The challenges in analyzing these pharmaceuticals include nucleic-acid analysis, protein structure and sequence, and degree of nucleic-acid incorporation. To characterize PTMs and impurities, enzymatic digestion is commonly used. Protease enzymes, such as trypsin and pepsin can be applied for this. In 2021, Toole *et al.* developed a rapid offline digestion method by using enzyme-immobilized beads coated with a thermally stable enzyme (*e.g.* trypsin and pepsin).^89^ In their study, the use of two different enzymes was compared. The authors concluded that trypsin is more favored for use than pepsin due to the longer digestion time. However, with the use of enzyme-immobilized beads, this can be more controlled, resulting in a digestion time of 30 minutes which is comparable with trypsin approaches. Nevertheless, implementing sample transformation in an online way has not been reported.^90^ A similar online IMER and reduction approach as applied in the field of mAbs could be promising for AAVs as well. The authors did critically note that the stability of AAVs and material limitations needs to be investigated in an online sample transformation platform.

#### 3.3.3 Solvent-induced transformation

In addition to thermal- and enzymatic transformation, the use of solvents has also been reported for the analysis of particle constituents. For NPs with encapsulated compounds, such as the API in medicinal NPs, the NP first needs to be disassembled to get all compounds in solution. Generically, this is performed by dissolving (*i.e.* disassembling) the NPs in an organic solvent prior to analysis.^78,91,92^ Rather than performing this step offline, the disassembly can also be integrated with an online interface. In 2010, Helle *et al.* presented an online LC system for dissolution (*i.e.* drug release) studies of polymeric NPs.^93^ The polymeric NPs were disassembled online with methanol after which the contents were analyzed offline by RPLC-DAD. To our knowledge, this was the first study in which online decomposition studies have been performed on NPs.

Rather than only measuring the constituents after disassembly, a first-dimension separation prior to disassembly can be integrated to study the intact nanoparticle as well. In 2017, we published an online hydrodynamic chromatography (HDC) × SEC method in which polymeric nanoparticles were separated on size in a first dimension and disassembled.^94,95^ Tetrahydrofuran (THF) was used as a disassembly solvent. Subsequently, the polymer was analyzed in a second dimension. This 2D-LC method demonstrates that by implementing disassembly as an online transformation, the correlation between two orthogonal sample dimensions can be obtained. Also, the online approach allowed for efficient NP analysis. The conventional analysis time was decreased as separate analysis steps were omitted. Moreover, no sample preparation was needed for the 2D-LC methods, allowing for direct analysis of the NP sample.

#### 3.2.4 Other transformation mechanisms

Chemical transformation is another aspect that can be implemented to study the effect of different chemical conditions on NP characteristics. In the case of environmental NPs, weathering is a term that describes the natural degradation of nanoplastics.^84,96^ The variety of weathering conditions is large, yet the most abundant transformations that naturally occur are biodegradation (*i.e.* (enzymatic) hydrolysis), (UV-photo-oxidation, chemical oxidation, and thermal degradation).^97^ Weathering conditions on environmental micro- and nanoplastics can be mimicked to predict aggregation or degradation of the NPs. However, Alimi and colleagues pointed out that efficient experiments to perform such experiments are still limited.^97^ Therefore, it is suggested that online sample transformation could be of great use in the field of weathering analyses.

For medicinal NPs, chemical sample transformation can be implemented to study the influence of temperature, light, pH, or other factors on the NP and API stability.^78,98,99^ DDSs can be used for the targeted administration of drugs on tumor cells as the pH in some tumor cells is much lower than in regular cells.^100^ Samanta and co-workers monitored the *in vitro* drug release of doxorubicin-loaded nanoparticles by diluting these in physiological pH and low pH and measuring the resulting signal with fluorescence spectroscopy.^96,101^ The acidification was performed offline without additional LC separation. Their acid-catalyzed hydrolysis occurred at a relatively low reaction rate, yet the exact reaction time was not reported.

**Fig. 5:**
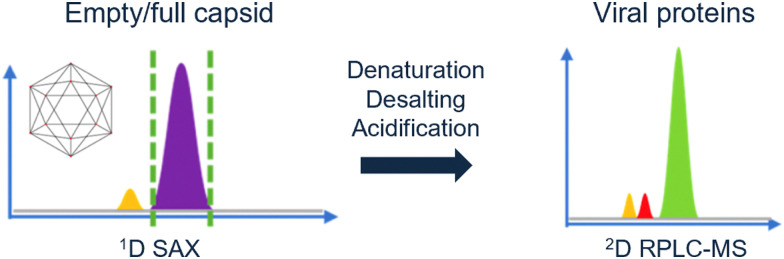
Schematic overview of the multi-attribute separation of AAVs with online acidification. Reprinted from Wu *et al.*, *Anal. Chem.*, 2022 under the CC-BY-NC-ND license. Copyright 2022 American Chemical Society.^103^

Likewise, Wu *et al.* demonstrated a 2D-LC method in AAVs were acidified online.^102^ With anion-exchange chromatography the full and empty capsids were separated. Next, the capsids were denatured and desalted by online acidification. The resulting viral proteins were then analyzed by RPLC-MS. The group of Wu demonstrated how pH transformation can be implemented in an automated 2D-LC set-up (Fig. 5), thereby characterizing multiple attributes in a single method.^103^

## 4. Synthetic polymers

### 4.1 Introduction

In contrast to natural polymers, synthetic polymers are classified as polymers derived from non-natural sources that are synthesized in a laboratory. The variety of synthetic polymers is immense, *e.g.* polyesters, polyacrylates, and polyurethanes. These polymers can be applied in many disciplines, such as packaging material, cloths, coatings, and implants. By definition, a polymer is composed of a strand of repeating units (*i.e.* the monomer).^104^ The polymers occur in different structures, such as linear, cyclic, branched, or cross-linked configurations. Similar to the occurrence of different peptides in a protein, synthetic polymers can also consist of different monomers, resulting in a copolymer. Likewise, copolymers also exist in different conformations; random, block, graft, or alternating arrangements. Regarding their complexity, different analytical methods are needed to characterize the polymer attributes.

### 4.2 Characterizing synthetic polymers

**Fig. 6:**
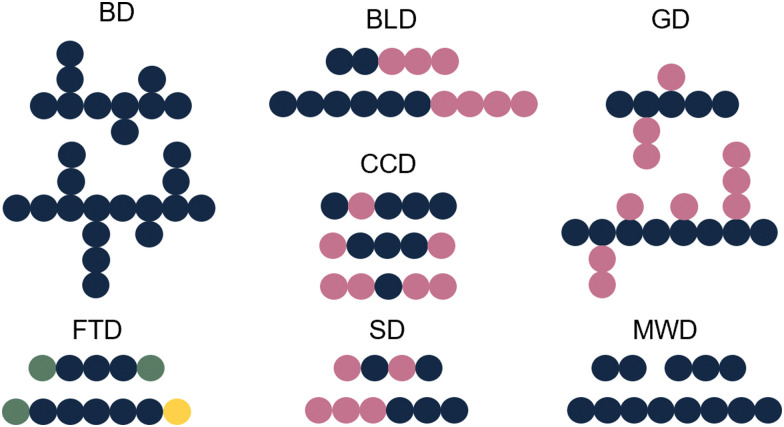
Schematic representation of different polymer distributions. Blue: monomer A, pink: monomer B, green: functional group C, yellow: functional group D.

Whilst sample transformations are already implemented in the field of biopolymers, it is less explored in the analysis of synthetic polymers. Similar to biopolymers, synthetic polymers are molecules that have rather complex compositions and often require multiple analytical techniques to analyze different sample attributes.^105^ Properties of interest include MW determination, polymer sequence, monomer ratios, blockiness (*i.e.* degree of adjacent monomers in copolymer block), branching, polydispersity index (PDI), and end-group identification.^105,106^ These properties are often not described as single values but rather as distributions of these specific characteristics. Examples include molecular-weight distribution (MWD), sequence distribution (SD), branching distribution (BD), chemical-composition distribution (CCD), grafting distribution (GD), functionality-type distribution (FTD), and the block-length distribution (BLD) (Fig. 6).^107^ All of these distributions are essential for the comprehensive characterization of the polymer composition, structure, and properties.

The shape and width of the MWD (also characterized by the PDI) influences the properties of the polymer and are therefore one of the key properties of interest.^108–110^ In the case of a polymer with monomers A and B, that could be copolymer A–A–B–A. Determination of the sequence of the comprising monomers is known as the SD. When different copolymers exist in a sample (*e.g.* A–A–B–A, B–A–B–B, B–A–A–B), the distribution of these different copolymer chains could also be determined, resulting in the CCD. Whilst the SD looks at the distribution of monomers inside a polymer chain, the CCD covers the distribution of the polymer chains with different compositions. Note that for CCD determination the SD determination per chain type is not needed; differences in monomer ratios are sufficient. Additionally, often the FTD is also of interest. For this property, the end group functionalities are described. In addition to the distributions, also blockiness and branching are reported.

The multi-attribute analysis of synthetic polymers has already been described in literature.^105,106,111^ 2D-LC approaches were reported that provide information on the different sample distributions, such as MWD, FTD, CCD, and BD. Most methods described the MWD and one other distribution. Despite the wide variety of existing 2D-LC methods for polymer separations, the implementation of sample transformation was not well applied.

### 4.3 Sample transformation for synthetic polymers

#### 4.3.1 Thermal transformation

MW reduction can simplify the characterization of properties such as monomer composition and monomer SD in synthetic polymers. From the starting polymer with a given (high) MW, smaller fragments or monomers are released. Few online implementations of sample transformation are reported in literature. As explained in the previous chapter, Py-GC-MS is one of the most established tools to study the monomer composition. Here, Py-GC-MS will only be discussed when hyphenated with another dimension.

**Fig. 7:**
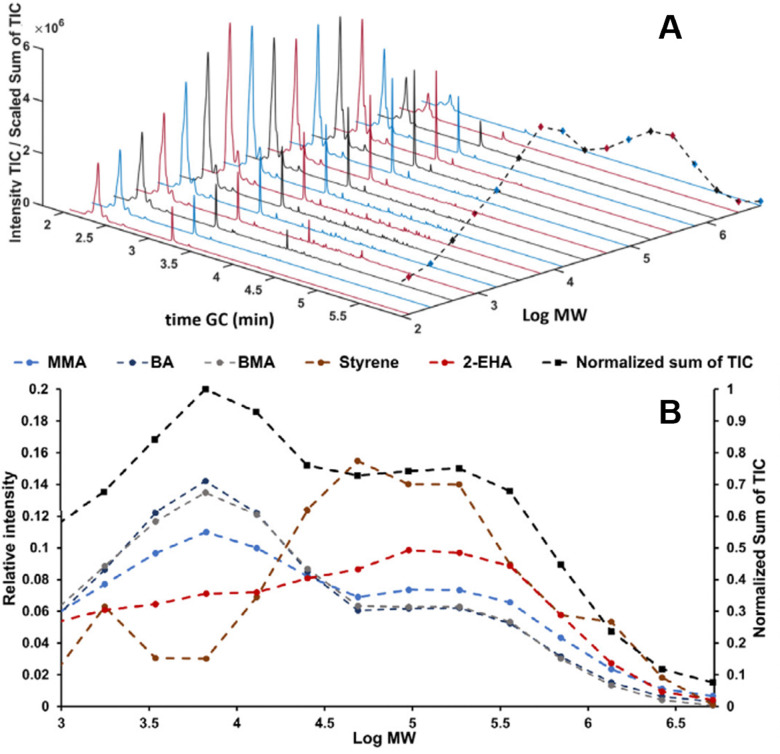
SEC-Py-GC-MS results, stacked plot of the obtained Py-GC runs the dashed black line traces the sum of the TIC (A). Relative distribution of monomers over MW of a complex copolymer *vs.* the chemical composition over the five different monomers present obtained from the Py-GC transformation (B). Adapted from Knol *et al.*, *J. Chromatogr. A*, 2023 under the CC-BY license.^113^

In recent studies, such couplings are reported by combining SEC with Py-GC-MS in the liquid phase. A unique combination of information on copolymer heterogeneity for styrene/acrylate copolymers was obtained by our group. Initially, the coupling was performed offline.^112^ Later the coupling was performed online.^113^ This analytical platform enabled a direct correlation between the MWD, CCD, and blockiness. Fig. 7 contains a demonstration of the analytical platform on a complex industrial copolymer containing five different monomers including styrene and various acrylates where the CCD is determined as a function of MW.

#### 4.3.2 Other transformation mechanisms

Besides pyrolysis for MW reduction of polymers, fragmentation inside the MS can also be used. Mengerink *et al.* demonstrated multiple fragmentation in the MS to study BLD of polyamide copolymers.^114^ The sample was first subjected to an LC separation and subsequently transformed (*i.e.* fragmented) by MS/MS to identify co-oligomeric fragments. In our group, Bos *et al.* studied the CCD of cellulose ethers by performing offline hydrolysis of the glycosidic bonds by acid hydrolysis.^115^ This resulted in substituted monomers which were analyzed by LC-MS to obtain the substitution degree and composition of β-glucose monomers.

Another approach to obtain the correlation between the MWD and CCD that does not utilize MW reduction is derivatization. This was demonstrated in our group by Brooijmans *et al.*^116^ In this work, characterization of the carboxylic acid FTD as a function of MWD was obtained by offline derivatization of the acid groups using a UV-absorbing agent and subsequently performing SEC using multiple selective detectors. Similarly, already in 2004, offline derivatization was used by Oudhoff *et al.* to characterize hydroxyl end groups in polyols.^117^ Hydroxyl end groups were converted to chargeable UV-absorbing groups to characterize mono- and difunctional byproducts in a trifunctional polyol sample by CE.

Aside from sample transformation aiding in the characterization of the polymer itself, sample transformation has sometimes been applied to study its transformation products (TPs) or degradation behavior. However, this was typically performed offline. One example in which online degradation was performed was earlier described in the previous chapter by using a lipase-functionalized IMER coupled to SEC for polymeric NPs.^118^

Offline approaches using enzyme-mediated degradation of polymers include application in DDSs.^119^ In the past, our group worked on offline degradation of poly(ester amide). In this example, a mixture of enzymes was used and the sample was incubated for four weeks. The same group later described an online system to characterize poly(ester amide) TPs.^120^ This system used a continuous infusion of enzyme solution with sample followed by the collection of the degradation products in a loop, subjecting it to LC-MS by switching a valve. In this online setup, reaction times below 15 minutes already demonstrated the formation of TPs. Other studies utilized abiotic (non-enzymatic) degradation by offline hydrolysis in a heated oven for several days.^121^ The authors subjected biodegradable poly(ester-urethane-urea) products to hydrolytic degradation followed by LC-MS analysis of the TPs to study possible TPs released into the environment. Similarly, hydrolytic degradation of stabilized poly(bisphenol A)carbonate was investigated for incubation times of up to 100 days followed by LC and 2D-LC measurements of the TPs.^122^ In the case of PLGA degradation, accelerated offline hydrolysis was demonstrated Pourasghar *et al.*^123^ The hydrolysis reaction as performed under alkaline conditions (0.1 M KOH) for 90 minutes at 100 °C followed by derivatization of the generated lactic and glycolic acid prior to LC-UV analysis. An alternative approach is using a specialized controlled-environment chamber to perform accelerated aging studies, for example on stabilized polypropylene.^124^ However, aging experiments still took several weeks, even at 135 °C, prior to LC-MS analysis of the TPs. In 2021, Wolf *et al.* used a custom-made thermal desorption oven to perform thermal oxidation on low-density polyethylene and collect the volatile oxidized oligomers.^125^ This degradation was performed at 225 °C and took 30 minutes. The TPs were then dissolved and subjected to offline NPLC and GC-MS.

Lastly, several studies investigated the photostability of various polymers including poly(ethylene terephthalate),^126^ poly(vinyl chloride) films,^127^ and polypropylene.^128^ These offline photodegradation studies were however quite time-consuming taking from several hours to months of aging time prior to analysis of TPs.

## 5. Small molecules

### 5.1 Introduction

Small molecules are ubiquitous and could be both natural or synthetic. They occur in a large number of conformations or shapes and with a broad spectrum of applications such as in food, pharmaceuticals, artworks, fragrances, polymers, and energy carriers. In the pharmaceutical industry, drug-discovery processes employ massive databases of, in some cases, over one million different small molecules that are synthesized and screened against a specific target for pharmaceutical activity.^129^ For the purpose of this review, anything smaller than roughly 1 kDa is considered a small molecule.

In the case of small molecules, analysis of the molecules themselves is relatively straightforward. Yet, sample transformation is interesting in several contexts such as aging of dyes, biodegradation in the environment, or studying the stability and metabolism of pharmaceuticals or food products.

### 5.2 Characterizing small molecules

Due to the large chemical variety of properties that small molecules may have, different separation modes are applied in modern analytical separation technology. LC offers a broad spectrum of separation modes based on chemical properties such as molecular size, charge, hydrophobicity, chirality, affinity, and saturation (double bonds). As also highlighted in the previous sections, 2D-LC is an upcoming technique that may be employed to characterize multiple properties in a single analytical platform. While MW reduction is ostensibly not useful for characterization, there are still scenarios in which sample transformation is of interest to small-molecule analysis. In our literature search, we focused in particular to include the study of the degradation of a molecule after exposure to certain conditions such as light, heat, or various stages of metabolism. Another aspect of sample transformation that is commonly applied to small molecules is derivatization. However, in this context, derivatization is often applied to improve the detectability of the analytes. For example, by adding a UV-absorbing or fluorescent group to a molecule to enable UV and/or fluorescence detection.^130^ Alternatively, a common approach is an isotope label for MS applications.^131^ In these cases, sample transformation is not directly used to obtain information about an analyte property and for this reason, will not be considered in this review. The interest in using sample transformation within a multi-dimensional setup originates from the necessity to characterize both the degradation pathway as well as the degradation products. One such degradation pathway is light-induced degradation. It is of interest to various fields including cultural heritage,^132^ food,^133^ pharmaceuticals,^134^ and polymers.^135^ In the field of cultural heritage, in which objects may be exposed to various conditions (*e.g.* light, temperature, humidity) throughout time, the organic colorants in these cultural-heritage objects might degrade.^136^ This results in a change of appearance over time. Conservation of the object requires knowledge about the chemical identity of the colorant and understanding the degradation pathways that occur when exposed to, for example, light. Besides degradation resulting from external factors like light and temperature, small drug molecules may undergo a chemical transformation as they get metabolized by various biological systems to aid in excretion from the body.^137^ Some chemical alterations that can occur in the human body include oxidation, hydrolysis, isomerization, and hydration.^138^ In most cases, metabolic alteration of the drug results in reduced pharmaceutical activity. However, it may also result in the generation of a pharmaceutically active metabolite. Therefore, it is important to understand the drug metabolism of a new drug entity before it is commercially available. Furthermore, the metabolism of certain small molecules present in the environment may also be of concern, such as polycyclic aromatic hydrocarbons (PAHs). PAHs originate from incomplete combustion of organic matter such as fuel.^139^ Exposure to PAHs results in severe health risks and therefore understanding possible transformations that may occur in the environment as well as during mammalian metabolism are important.

### 5.3 Sample transformation for small-molecule analysis

While various forms of sample transformation are of interest for small molecules as highlighted above, literature on implementations of such transformations into analytical platforms is scarce. As mentioned before, derivatization is commonly used for better detectability. Yet, examples exist in which derivatization was used to aid in structure elucidation.^140^ To cover a few examples, it could be used to identify the position of the C=C bond in unsaturated fatty acids by LC-MS.^141^ Derivatization has also been used to distinguish primary amines from other amino acids.^142^ Another approach is hydrogen/deuterium exchange, which is used to probe the number of exchangeable hydrogens. This is commonly coupled with MS-based detection.^143^ Recently, it has been performed online in a 2D-LC setup using heavy water and an aprotic organic solvent for structure elucidation of drug metabolites.^144^ Moverover, advances have been made in the field of light-induced and electrochemical transformations. In our group, the first online implementation of light-induced transformation in 2D-LC has been demonstrated. Therefore, we will discuss online applications of both light-induced and electrochemical transformations in separation platforms in more detail.

#### 5.3.1 Light-induced transformation

**Fig. 8:**
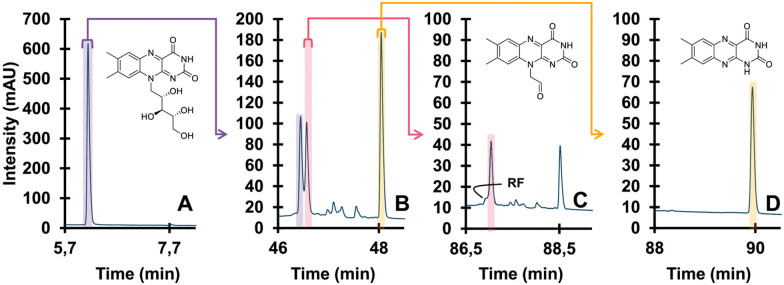
Detailed degradation study of riboflavin. Undegraded (A), degraded (B), isolated TP (pink) and further degraded (C), different isolated TP (yellow) and further degraded (D). Reprinted from den Uijl *et al.*, *J. Chromatogr. A*, 2023 under the CC-BY license.^152^

In a light-induced degradation study by Pirok *et al.*, the degradation experiment was performed offline and the degradation products were subsequently analyzed by LC.^136^ In this study, our colleagues also highlight the importance of the conditions used for light degradation. To throw light on our work, our group published multiple studies that implemented light degradation in LC platforms. Groeneveld *et al.* extensively reviewed the influence of the solvent (*e.g.* presence of dimethyl sulfoxide (DMSO)), wavelength, temperature, and presence of oxygen on the rate of photodegradation and its mechanism.^145^ In 2021, a liquid–core waveguide (LCW) was used by our group to study the chemistry of light-induced degradation, which may be implemented online.^132^ The described LCW utilizes the concept of total internal reflection for efficient illumination of the sample and was made from gas-permeable material to permit diffusion of air into the cell during light exposure. The implementation of an LCW in an analytical separation system was already reported in 1999 for the purpose of Raman detection after an LC separation.^146,147^ The use of an LCW as a photochemical reactor has recently been reviewed by our group.^148^ Shortly after, our group demonstrated the first online coupling of an LCW to an analytical separation system to study light-induced degradation.^149^ Following upon this work, we demonstrated the implementation of an LCW in (mD-)LC systems in several recent works.^150–152^ The initial system used a multiple heart-cutting approach where a mixture of analytes can be separated in the first dimension and several analytes may be selected to be subjected to light degradation.^151^ The degradation products can subsequently be analyzed by a second-dimension separation. Another configuration was a recycling LC system which enabled a detailed study of the multi-stage light-degradation pathway in an automated fashion (Fig. 8).^152^ Besides automation, the design of the LCW facilitated accelerated light degradation and an irradiation time of only ten minutes was sufficient. When comparing to offline degradation studies, degradation times of up to 80 hours may be required whereas in the LCW degradation was observed within one hour.^150^ While these works demonstrate the power of online light degradation for cultural heritage applications, it could also be implemented in different areas such as food and pharmaceuticals.^152^

#### 5.3.2 Electrochemical transformation

Another form of sample transformation that has been studied for several decades has been electrochemistry (EC) and its coupling with LC and/or MS.^153^ EC is a versatile technique that may be used to perform all sorts of reactions but is most commonly utilized for oxidation reactions. Electrochemical oxidation of small organic compounds can be used to simulate drug metabolism in the human body.^154,155^ Pharmaceuticals are typically metabolized by enzymes in the liver that are part of the cytochrome P450 family to increase their polarity, enabling faster excretion. In 2012, Jahn and Kart published an extensive review on the state and future perspectives of EC coupled to LC-MS or directly to MS.^153^ In some cases, LC was not required, however, it was possible that EC produces a mixture of isomeric or isobaric molecules that require separation prior to MS detection.^156^ For the purpose of this review, we will only highlight more recent publications of EC hyphenated with LC(-MS). One of the large application areas for EC-based sample transformation is in the pharmaceutical industry for drug development.^155^ EC can be used for both simulating the human metabolism as well as simulating TPs formed during the drug shelf-life (*i.e.* aging). For instance, Torres *et al.* compared offline and online EC-based oxidative stability studies of API in solution.^157^ The online system facilitated automatic sequential analyses of a number of varied conditions without operator intervention. With regard to drug metabolism studies, the EC can be placed either before or after the LC separation. One application demonstrating the utility of LC-EC-MS was to first separate cytokinins by LC and subsequently study the oxidation products of selected cytokinins to MS.^158^ Several studies demonstrated the use of EC to simulate both phase I and phase II human metabolism.^159,160^ Phase I metabolism was simulated by EC-based oxidation while phase II was simulated by the addition of glutathione for follow-up reactions. Several studies investigated phase I and phase II metabolism of selected cardiovascular drugs^159,161^ and immunosuppressants^162^ by EC-LC-MS. Moreover, similar EC-LC-MS systems have been employed for the prediction of TPs of the fungicide fluopyram^163^ and the hormone thyroxine.^164^ For food applications, a 2019 review by Sontag *et al.* describes electrochemical methods to analyze easily oxidizable analytes such as phenolics, pesticides, or vitamins.^165^ However, the authors also discuss approaches for analytes exhibiting poor electrochemical activity by transformation to electroactive substances using pre-column derivatization. One of the compounds studied is vitamin D and its metabolites using EC.^166^ The authors stated that this technology can support or even replace animal studies in the future. Another study transformed five citrus flavonoids using EC to simulate phase I and phase II metabolism.^167^ Knoche *et al.* studied the possible TPs of lasalocid, an antibiotic and growth-promoting feed additive used in cattle and poultry farming.^168^ The authors compared EC and a liver microsome assay both followed by LC-MS. The EC was concluded to be time and cost-saving as no costly enzymes or microsomes are needed as well as no incubation time (90 minutes). However, EC was not able to simulate all microsomal transformation reactions. Besides metabolism-related studies, Zheng *et al.* applied offline EC-LC-MS/MS to study the flavor formation mechanism in aged liquors.^169^ Environmental degradation pathways are also mimicked using EC coupled to LC-MS. This facilitates the elucidation of oxidative degradation pathways of molecules of agrochemical interest (*e.g.* pesticides) in the environment.^170^ The organophosphate insecticide chlorpyrifos is a pesticide that was studied by EC-LC-MS.^171^ Busy *et al.* used EC-LC-MS to study the reductive metabolism of 3-trifluoromethyl-4-nitrophenol, a pesticide used for population control of fish in lakes.^172^ EC was also applied to study oxidative TPs of PAHs to understand both biological and environmental degradation behavior.^173^ Similarly, the technique was applied to study TPs of carbamazepine generated by white-rot fungus.^174^ The authors report that the combination of EC-LC-MS was more effective than software tools to define screening targets and faster than non-targeted TP screening.

## 6. Conclusions

Sample transformation has opened up new opportunities for the determination of, and correlation to, sample dimensions that would previously be difficult to analyze. The applications covered in this work demonstrate that the chemical transformation of samples prior to or during analysis paves the way to more detailed structural elucidation and stability studies. There are a number of conclusions to be drawn.

Firstly, the transformation mechanisms that we found can be classified as (i) selective reduction of molecular weight (*e.g.* enzyme-mediated depolymerization/digestion of proteins), (ii) destruction; breaking covalent bonds of molecules with low selectivity (*e.g.* pyrolysis of polymers) (iii) decomposition, altering non-covalent interaction of sample components (*e.g.* solvent-mediated disassembly of nanoparticles) and (iv) derivatization, reveal the presence of specific chemical groups or motif in part of the sample (*e.g.* group type functionalization for polymers).

Secondly, for protein and nucleic-acid therapeutics, multilevel analytical strategies with sample transformation allow the study of specific portions of the large molecules. The workflows covered in this review mainly rely on mechanisms that selectively reduce the molecular weight and/or sample complexity by altering or removing post-translational or transcriptional modification.

Thirdly, the works covered in this review also underline the power of implementing sample transformation for NP analysis. It not only automates initially labor-intensive analyses but also provides more information on the sample. Indeed, insight into the correlation between sample dimensions is obtained that would not have been possible with only intact NP analysis. However, sample transformation is by no means as widely applied in the field of NPs yet relative to protein and nucleic-acid therapeutics.

Fourthly, while only reported by a limited number of studies, the online sample transformation has been demonstrated in the field of synthetic polymers. By this approach, information on the chemistry and/or its TPs was obtained under various conditions. The large portion of literature using offline sample transformation to study polymers in a timely and often non-automated manner highlights the importance of sample transformation and potential future improvements using online implementations of sample transformation to study polymers. We foresee that interdisciplinary collaborations will enhance this implementation. As an example, we expect the development of enzymes capable of selectively cleaving bonds to achieve characterization similar to the field of biopolymers.

Finally, multiple studies described above concluded that online implementations of light-induced degradation and EC-based transformation resulted in the accelerated transformation of small molecules without the need for manual intervention or expensive microsomes.

Overall, the combination of transformation reactors and analytical methods is yielding exciting new avenues to characterize contemporary samples. The incorporation of sample transformation reduces sample complexity for easier characterization, while simultaneously unlocking new sample dimensions for complete characterization of complex samples. Looking onward, we anticipate that the continued push in separation science to construct multi-dimensional separation systems will aid in the coupling of transformation reactors. We thus envision an increase in applications capitalizing on these developments. We also foresee a greater role for chemometric analysis and computer-based method development to distill useful information and trends from these complex datasets.

## Author contributions

Annika A. M. van der Zon: investigation, visualization, writing – original draft. Joshka Verduin: investigation, visualization, writing – original draft. Rick S. van den Hurk: investigation, visualization, writing – original draft. Andrea F. G. Gargano: supervision, funding acquisition, writing – review & editing. Bob W. J. Pirok: supervision, funding acquisition, writing – review & editing.

## Conflicts of interest

There are no conflicts to declare.

## Supplementary Material

CC-060-D3CC03599A-s001
