# Supplementary material for: Sample transformation in online separations: how chemical conversion advances analytical technology
Source: Chem Commun (Camb). 2023 Nov 15;60(1):36–50. doi: 10.1039/d3cc03599a (PMC10729587; doi:10.1039/d3cc03599a)
Supplement: CC-060-D3CC03599A-s001 [file CC-060-D3CC03599A-s001.pdf]

This journal is © The Royal Society of Chemistry 2023

ELECTRONIC SUPPLEMENTARY INFORMATION

Sample transformation in online separations: how chemical conversion advances analytical technology

Annika A.M. van der Zon [† a, b], Joshka Verduin [† b, c], Rick S. van den Hurk [† a, b], Andrea F.G. Gargano [a, b], Bob W.J. Pirok [a, b, *]

[a] University of Amsterdam, van 't Hoff Institute for Molecular Sciences (HIMS), Analytical Chemistry Group, Science Park 904, 1098 XH Amsterdam, the Netherlands

[b] Centre of Analytical Sciences Amsterdam (CASA), Science Park 904, 1098 XH Amsterdam, the Netherlands

[c] Vrije Universiteit Amsterdam, Department of Chemistry and Pharmaceutical Sciences, Amsterdam Institute of Molecular and Life Sciences (AIMMS), Division of BioAnalytical Chemistry, De Boelelaan 1085, 1081 HV, Amsterdam, the Netherlands

[†] These authors contributed equally

* Corresponding author

Bob W.J. Pirok B.W.J.Pirok@uva.nl

Contents

S-1 Overview of the described applications 3

References 7

S-1 Overview of the described applications

Table S1 summarizes the applications described in the manuscript based on the sample type (*e.g.* nanoparticles, polymers, or small molecules). For each sample type, the property of interest is highlighted along with the sample transformation that is applied, the approach used, the type of coupling, and the analytical technique. All abbreviations used in the table are described on the below the table.

Table S1: Overview of the properties of interest of the different sample types and its corresponding sample transformation and analytical method.

Sample type	Property of interest	Sample transformation	Approach	Coupling	Technique	Ref
**Antibodies**						
mAb and bsAb	Charge variants, deamidation, oxidation, isomerization, peptide mapping	MW reduction	Enzymatic (Lys-C, trypsin), reduction (TCEP)	Online	mD-LC-MS	1
mAb	Peptide sequencing	MW reduction	Enzymatic (trypsin)	Online	mD-LC-MS	2
mAb	Deamidation, isomerization, succinimide, peptide mapping	MW reduction	Enzymatic (trypsin), reduction (DTT or TCEP)	Online	mD-LC-MS	3
mAb	Charge variants, deamidation, isomerization, glycosylation, peptide mapping	MW reduction	Enzymatic (trypsin), reduction (DTT)	Online	mD-LC-MS	4
mAb	Oxidation, succinimide, deamidation or glycosylation (without peptide mapping)	MW reduction	Enzymatic (trypsin), reduction (TCEP)	Online	mD-LC-MS	5
mAb	Glycosylation	MW reduction	Enzymatic (IdeS), reduction (TCEP)	Online	mD-LC-MS	6
mAb	Deamidation, oxidation, isomerization, glycosylation, peptide mapping	MW reduction	Enzymatic (IdeS, trypsin), reduction (TCEP)	Online	mD-LC-MS	7
mAb	Deamidation, oxidation, glycation, peptide mapping	MW reduction	Enzymatic (trypsin), reduction (DTT)	Online	mD-LC-MS	8
mAb and bsAb	Deamidation, oxidation, glycation, glycosylation, peptide mapping	MW reduction	Enzymatic (trypsin), reduction (DTT)	Online	mD-LC-MS	8
bsAb	Charge variants, size variants, peptide mapping	MW reduction	Enzymatic (PNGase F, trypsin, Lys-C), reduction (gingiskhan, TCEP, DTT)	Online	LC-MS	9
mAb and bsAb	Charge variants, deamidation, oxidation, isomerization, peptide mapping	MW reduction	Enzymatic (trypsin), reduction (TCEP)	Online	mD-LC-MS	10
mAb	Size variants, sequence variants, aggregates, glycosylation, glycation,	MW reduction	Enzymatic (trypsin)	Online	mD-LC-MS	11
bsAb	Charge variants, deamidation, proline amidation, oxidation,	MW reduction	Enzymatic (trypsin), reduction (DTT)	Online	mD-LC-MS	12

	isomerization, succinimide, peptide mapping					
mAb	Charge variants, deamidation, N-terminal glutamine cyclization, oxidation, proline amidation, peptide mapping	MW reduction	Enzymatic (trypsin)	Online	mD-LC-MS	13
mAb	Peptide mapping	MW reduction	Enzymatic (trypsin)	Online	CZE-DAD	14
mAb	Peptide mapping	MW reduction	Enzymatic (trypsin)	Online	CZE-DAD	15
mAb	Peptide mapping	MW reduction	Enzymatic (trypsin), offline-reduction (DTT)	Online	CZE-DAD	16
mAb	Fragment/peptide mapping	MW reduction	Enzymatic (IdeS)	Online	CZE-MS	17
mAb	Charge variants, deamidation, oxidation, peptide mapping	MW reduction	Enzymatic (Pepsin), reduction (TCEP)	Online	2D-CZE-MS	18
**ADC**						
ADC	Size variants, oxidation, deamidation, aggregates	MW reduction	Enzymatic (trypsin), reduction (TCEP)	Online	mD-LC-MS	19
**Fc-fusion protein**						
mAb and Fc-fusion protein	Deamidation, succinimide formation, oxidation, C-terminal lysine clipping, N-terminal glutamine cyclization, glycosylation, proline amidation	MW reduction	Enzymatic (trypsin, Lys-C)	Online	mD-LC-MS	20
mAb and Fc-fusion protein	Oxidation, succinimide, deamidation, isomerization, glycosylation, peptide mapping	MW reduction	Enzymatic (trypsin), reduction (TCEP)	Online	mD-LC-MS	21
**Nucleic acid**						
sgRNA	Nucleotide mapping	MW reduction	Enzymatic (RNase T1, A, U2)	Online	mD-LC-MS	22
gRNA	Nucleotide mapping	MW reduction	Enzymatic (RNase T1)	Online	LC-MS	23
**Nanoparticles**						
Polymeric (acrylic)	Multi-attribute characterization (size, MW)	Decomposition (disassembly)	Solvent (organic)	Online	HDC×SEC	24
Polymeric (acrylic)	Multi-attribute characterization (size, MW)	Decomposition (disassembly)	Solvent (organic)	Online	HDC×SEC	25
Medicinal (AAV)	Multi-attribute characterization (empty/full, NP components)	Decomposition (disassembly)	Chemical (pH)	Online	AEX-RPLC	26
Medicinal (AAV)	Multi-attribute characterization(peptide mapping)	MW reduction	Enzymatic (trypsin, pepsin)	Offline	LC-MS/MS	27
Medicinal/polymeric (PLGA)	Protein adsorption	Decomposition (desorption), MW reduction	Reduction (DTT), enzymatic (trypsin)	Offline	RPLC-MS/MS	28
Medicinal (polymer)	Drug release	Decomposition (dissolution)	Chemical (pH)	Offline	no LC (DLS, TEM, FLR)	29
Environmental	Polymer quantitation	MW reduction	Thermal	Online	PyGC-MS	30
Medicinal/polymeric (ester)	Drug release	Decomposition (dissolution)	Solvent	Online	RPLC-UV	31
Polymeric	Polymer degradation (with microfluidic chip)	MW reduction	Enzymatic (Novozym 51032 cutinase)	Offline	RPLC-MS	
Polymeric (polyester)	Polymer degradation (with IMER)	MW reduction	Enzymatic (lipase)	Online	SEC-ELSD	32
**Synthetic polymers**						
Copolymers (styrene/acrylate)	MWD, CCD, blockiness	MW reduction	Thermal	Offline	SEC-Py-GC-MS	33

Homopolymers (styrene) and copolymers (styrene/acrylate)	MWD, CCD, blockiness	MW reduction	Thermal	Online	SEC-Py-GC-MS	34
Copolymers (amide)	BLD	MW reduction	CID	Online	LC-CID-MS/MS	35,36
Copolymers (carboxylic acid)	MWD, CCD	Derivatization	Chemical	Offline	SEC-dRI-UV	36
Copolymers (alcohol)	FTD (end-groups)	Derivatization	Chemical	Offline	CE-UV	37
Medicinal NPs/copolymers (ester)	TPs	Destruction	Enzymatic (lipase)	Online	SEC-ELSD	32
Copolymers (ester/amide)	MWD	MW reduction	Enzymatic (α-chymotrypsin, proteinase K)	Offline	SEC-MS	38
Copolymers (ester/amide)	TPs	Destruction	Enzymatic (α-chymotrypsin)	Online	SEC-MS	39
Copolymers(ester/urethane/urea)	MWD, TPs	MW reduction, destruction	Enzymatic (urease), chemical (hydrolysis)	Offline	SEC-RID, RPLC-MS	40
Homopolymer (bisphenol A carbonate)	TPs	Destruction	Chemical (hydrolysis)	Offline	LCCC/SEC-UV/IR/MS, LCCC×SEC	41
Copolymer (ester)	TPs	Destruction	Chemical (hydrolysis)	Offline	RPLC-UV	42
Homopolymer (propylene)	TPs	Destruction	Chemical, accelerated-aging chamber	Offline	RPLC-UV-MS	43
Homopolymer (ethylene)	TPs	Destruction	Thermal oxidation	Offline	NPLC-UV, GC×GC-MS	44
Copolymer (ethylene/terephthalate)	photostability	Destruction	Photodegradation	Offline	IR	45
Homopolymer (vinyl chloride)	photostability	Destruction	Photodegradation	Offline	IR, viscometry	46
Homopolymer (propylene)	photostability, weathering	Destruction	Photodegradation	Offline	IR	47
**Small molecules**						
Cultural heritage (synthetic dyes)	TPs (photodegradation)	Destruction	Photodegradation	Offline	LC-MS	24
Cultural heritage (synthetic dyes)	TPs (photodegradation)	Destruction	Photodegradation	Offline	LC-UV	48
Cultural heritage (synthetic dyes)	TPs (photodegradation)	Destruction	Photodegradation	Online	LC-UV	49
Cultural heritage (synthetic dyes)	TPs (photodegradation)	Destruction	Photodegradation	Both	LC-UV	50
Cultural heritage (synthetic dyes)	TPs (photodegradation)	Destruction	Photodegradation	Online	2D-LC-UV	51
Cultural heritage (synthetic dyes)	TPs (photodegradation)	Destruction	Photodegradation	Online	recycling-LC-UV	52
Pharmaceuticals (R&D drug)	TPs (phase I metabolism)	Destruction	Electrochemistry	Both	EC-LC-UV-MS	53
Pharmaceuticals (cytokinins)	TPs (phase I metabolism)	Destruction	Electrochemistry	Online	LC-EC-MS	54
Pharmaceuticals (cardiovascular drugs)	TPs (phase I and II metabolism)	Destruction	Electrochemistry	Online	EC-LC-MS	55
Pharmaceuticals (cardiovascular drugs)	TPs (phase I metabolism)	Destruction	Electrochemistry	Online	EC-LC-MS	56
Pharmaceuticals (immunosuppressants)	TPs (phase I and II metabolism)	Destruction	Electrochemistry	Online	EC-LC-MS	57
Pharmaceuticals (fluopyram)	TPs (phase I metabolism)	Destruction	Electrochemistry	Online	EC-LC-MS	58
Pharmaceuticals (thyroxine)	TPs (phase I metabolism)	Destruction	Electrochemistry	Online	EC-LC-MS	59
Food (vitamin D)	TPs (phase I and II metabolism)	Destruction	Electrochemistry	Offline	LC-UV-MS	60
Food (citrus flavonoid)	TPs (phase I and II metabolism)	Destruction	Electrochemistry	Offline	LC-MS	61
Food (lasalocid antibiotic)	TPs (phase I metabolism)	Destruction	Electrochemistry, enzymatic	Offline	LC-UV-MS	62
Food (aged liquors)	TPs (aging)	Destruction	Electrochemistry	Offline	LC-MS	63
Environmental (herbicides)	TPs (environmental degradation)	Destruction	Electrochemistry	Offline	LC-MS	64
Environmental (insecticide)	TPs (phase I metabolism)	Destruction	Electrochemistry	Online	EC-LC-MS	65

Environmental (pesticide)	TPs (reductive metabolism)	Destruction	Electrochemistry	Offline	LC-MS	66
Environmental (PAHs)	TPs (metabolism and photooxidation)	Destruction	Electrochemistry, photodegradation	Both	EC-LC-MS	67
Environmental (pollutant)	TPs (phase I and II metabolism)	Destruction	Electrochemistry	Offline	EC-LC-MS	68

Adeno-associated virus (AAV), anion-exchange chromatography (AEX), antibody-drug conjugate (ADC), bispecific antibodies (bsAb), bond length distribution (BLD), capillary zone electrophoresis (CZE), chemical-composition distribution (CCD), collision-induced dissociation (CID), diode array detection (DAD), dithiothreitol (DTT), dynamic light scattering (DLS), electrochemistry (EC), evaporative light scattering detection (ELSD), fluorescence detection (FLR), functionality type distribution (FTD), gas chromatography (GC), guide ribonucleic acid (gRNA), hydrodynamic chromatography (HDC), immobilized-enzyme reactor (IMER), infrared (IR), liquid chromatography (LC), liquid chromatography at critical conditions (LCCC), mass spectrometry (MS), molecular weight (MW), molecular-weight distribution (MWD), monoclonal antibodies (mAb), multi-dimensional (mD), normal-phase liquid chromatography (NPLC), polyaromatic hydrocarbons (PAHs), poly(lactic-co-glycolic acid) (PLGA), pyrolysis (Py), refractive index detection (RID), research and development (R&D), reversed-phase liquid chromatography (RPLC), ribonuclease (RNase), ribonucleic acid (RNA), signal guide ribonucleic acid (sgRNA), size-exclusion chromatography (SEC), transformation product (TP), transmission electron microscopy (TEM), tris(2-carboxyethyl)phosphine (TCEP), two-dimensional (2D), ultraviolet detection (UV).

References

1 S. Oezipek, S. Hoelterhoff, S. Breuer, C. Bell and A. Bathke, *Anal Chem*, 2022, **94**, 8136–8145.

2 L. M. H. Reinders, M. D. Klassen, T. Teutenberg, M. Jaeger and T. C. Schmidt, *Anal Bioanal Chem*, 2021, **413**, 7119–7128.

3 J. Camperi, I. Grunert, K. Heinrich, M. Winter, S. Özipek, S. Hoelterhoff, T. Weindl, K. Mayr, P. Bulau, M. Meier, M. Mølhøj, M. Leiss, D. Guillarme, A. Bathke and C. Stella, *Talanta*, 2021, **234**, 122628.

4 L. Verscheure, A. Cerdobbel, P. Sandra, F. Lynen and K. Sandra, *J Chromatogr A*, 2021, **1653**, 462409.

5 J. Camperi, L. Dai, D. Guillarme and C. Stella, *Anal Chem*, 2020, **92**, 8506–8513.

6 J. Camperi, L. Dai, D. Guillarme and C. Stella, *Anal Chem*, 2020, **92**, 4357–4363.

7 J. Camperi, D. Guillarme and C. Stella, *Anal Chem*, 2020, **92**, 13420–13426.

8 S. Pot, C. Gstöttner, K. Heinrich, S. Hoelterhoff, I. Grunert, M. Leiss, A. Bathke and E. Domínguez-Vega, *Anal Chim Acta*, 2021, **1184**, 339015.

9 K. Mayr, T. Weindl, A. Gärtner, J. Camperi, T. Maetzke, M. Förster, T. Nachtigall, F. Steiner, A. Vogt, F. Hosp and M. Mølhøj, *Anal Chem*, 2023, **95**, 3629–3637.

10 A. Goyon, L. Dai, T. Chen, B. Wei, F. Yang, N. Andersen, R. Kopf, M. Leiss, M. Mølhøj, D. Guillarme and C. Stella, *J Chromatogr A*, 2020, **1615**, 460740.

11 F. Kuhne, K. Heinrich, M. Winter, J. Fichtl, G. Hoffmann, F. Zähringer, K. Spitzauer, M. Meier, T. A. Khan, L. Bonnington, K. Wagner, J. O. Stracke, D. Reusch, H. Wegele, M. Mormann and P. Bulau, *Anal Chem*, 2023, **95**, 2203–2212.

12 I. Grunert, K. Heinrich, M. Hingar, J. Ernst, M. Winter, K. Bomans, K. Wagner, A. Fevre, D. Reusch, M. Wuhrer and P. Bulau, *J Am Soc Mass Spectrom*, 2022, **33**, 2319–2327.

13 Z. Liu, Y. Cao, L. Zhang, Y. Xu and Z. Zhang, *Anal Chem*, 2023, **95**, 7977–7984.

14 Y. Ladner, S. Mas, G. Coussot, J. Montels and C. Perrin, *Talanta*, 2019, **193**, 146–151.

15 Y. Ladner, S. Mas, G. Coussot, K. Bartley, J. Montels, J. Morel and C. Perrin, *J Chromatogr A*, 2017, **1528**, 83–90.

16 M. Dadouch, Y. Ladner, C. Bich, J. Montels, J. Morel and C. Perrin, *Electrophoresis*, 2021, **42**, 1229–1237.

17 M. Dadouch, Y. Ladner, C. Bich, M. Larroque, C. Larroque, J. Morel, P.-A. Bonnet and C. Perrin, *Analyst*, 2020, **145**, 1759–1767.

18 J. Schlecht, K. Jooß, B. Moritz, S. Kiessig and C. Neusüß, *Anal Chem*, 2023, **95**, 4059–4066.

19 A. Goyon, M. Kim, L. Dai, C. Cornell, F. Jacobson, D. Guillarme and C. Stella, *Anal Chem*, 2019, **91**, 14896–14903.

20 J. Camperi, S. Dahotre, D. Guillarme and C. Stella, *Talanta*, 2022, **246**, 123519.

21 S. Dahotre, L. Dai, K. Kjenstad, C. Stella and J. Camperi, *J Chromatogr A*, 2022, **1672**, 463067.

22 A. Goyon, B. Scott, K. Kurita, C. Maschinot, K. Meyer, P. Yehl and K. Zhang, *Anal Chem*, 2022, **94**, 1169–1177.

23 A. Goyon, D. Nguyen, S. Boulanouar, P. Yehl and K. Zhang, *Anal Chem*, 2022, **94**, 16960–16966.

24 B. W. J. Pirok, G. Moro, N. Meekel, S. V. J. Berbers, P. J. Schoenmakers and M. R. van Bommel, *J Cult Herit*, 2019, **38**, 29–36.

25 B. W. J. Pirok, N. Abdulhussain, T. Aalbers, B. Wouters, R. A. H. Peters and P. J. Schoenmakers, *Anal Chem*, 2017, **89**, 9167–9174.

26 Z. Wu, H. Wang, A. Tustian, H. Qiu and N. Li, *Anal Chem*, 2022, **94**, 3219–3226.

27 E. N. Toole, C. Dufresne, S. Ray, A. Schwann, K. Cook and A. R. Ivanov, *Anal Chem*, 2021, **93**, 10403–10410.

28 H. Spreen, C. Barth, L. Keuter, D. Mulac, H.-U. Humpf and K. Langer, *J Drug Deliv Sci Technol*, 2022, **74**, 103543.

29 P. Samanta, K. Kapat, S. Maiti, G. Biswas, S. Dhara and D. Dhara, *J Colloid Interface Sci*, 2019, **555**, 132–144.

30 H. A. Leslie, M. J. M. van Velzen, S. H. Brandsma, A. D. Vethaak, J. J. Garcia-Vallejo and M. H. Lamoree, *Environ Int*, 2022, **163**, 107199.

31 A. Helle, S. Hirsjärvi, L. Peltonen, J. Hirvonen, S. K. Wiedmer and T. Hyötyläinen, *J Pharm Biomed Anal*, 2010, **51**, 125–130.

32 B. Wouters, B. W. J. Pirok, D. Soulis, R. C. Garmendia Perticarini, S. Fokker, R. S. van den Hurk, M. Skolimowski, R. A. H. Peters and P. J. Schoenmakers, *Anal Chim Acta*, 2019, **1053**, 62–69.

33 W. C. Knol, T. Gruendling, P. J. Schoenmakers, B. W. J. Pirok and R. A. H. Peters, *J Chromatogr A*, 2022, **1670**, 462973.

34 W. C. Knol, J. P. H. Smeets, T. Gruendling, B. W. J. Pirok and R. A. H. Peters, *J Chromatogr A*, 2023, **1690**, 463800.

35 Y. Mengerink, H. Philipsen, J. Jordens, J. Mengerink, R. van der Hoeven and R. A. H. Peters, *J Appl Polym Sci*, , DOI:10.1002/app.53683.

36 T. Brooijmans, R. A. Okhuijsen, G. M. M. Oerlemans, B. W. J. Pirok, P. J. Schoenmakers and R. A. H. Peters, *Anal Chim Acta*, 2019, **1072**, 87–94.

37 K. A. Oudhoff, F. A. VanDamme, E. P. C. Mes, P. J. Schoenmakers and W. Th. Kok, *J Chromatogr A*, 2004, **1046**, 263–269.

38 A. Ghaffar, G. J. J. Draaisma, G. Mihov, A. A. Dias, P. J. Schoenmakers and Sj. van der Wal, *Biomacromolecules*, 2011, **12**, 3243–3251.

39 A. Ghaffar, G. J. J. Draaisma, G. Mihov, P. J. Schoenmakers and Sj. van der Wal, *J Chromatogr A*, 2013, **1286**, 29–40.

40 C. K. Borrowman, M. Bücking, B. Göckener, R. Adhikari, K. Saito and A. F. Patti, *Polym Degrad Stab*, 2020, **178**, 109218.

41 L. Coulier, E. R. Kaal and Th. Hankemeier, *Polym Degrad Stab*, 2006, **91**, 271–279.

42 M. Pourasghar, A. Koenneke, P. Meiers and M. Schneider, *J Pharm Anal*, 2019, **9**, 100–107.

43 S. Beißmann, M. Stiftinger, K. Grabmayer, G. Wallner, D. Nitsche and W. Buchberger, *Polym Degrad Stab*, 2013, **98**, 1655–1661.

44 N. Wolf, S. Säger, M. Lommatzsch and T. J. Simat, *Polym Degrad Stab*, 2021, **185**, 109490.

45 G. J. M. Fechine, P. A. Christensen, T. A. Egerton and J. R. White, *Polym Degrad Stab*, 2009, **94**, 234–239.

46 E. Yousif, N. Salih and J. Salimon, *J Appl Polym Sci*, 2011, **120**, 2207–2214.

47 Z. An, Z. Xu, Y. Ye and R. Yang, *Anal Chim Acta*, 2021, **1169**, 338632.

48 I. Groeneveld, S. E. Schoemaker, G. W. Somsen, F. Ariese and M. R. van Bommel, *Analyst*, 2021, **146**, 3197–3207.

49 I. Groeneveld, I. Bagdonaite, E. Beekwilder, F. Ariese, G. W. Somsen and M. R. van Bommel, *Anal Chem*, 2022, **94**, 7647–7654.

50 M. J. den Uijl, A. Lokker, B. van Dooren, P. J. Schoenmakers, B. W. J. Pirok and M. R. van Bommel, *Dyes and Pigments*, 2022, **197**, 109882.

51 M. J. den Uijl, Y. J. H. L. van der Wijst, I. Groeneveld, P. J. Schoenmakers, B. W. J. Pirok and M. R. van Bommel, *Anal Chem*, 2022, **94**, 11055–11061.

52 M. J. den Uijl, I. Bagdonaite, P. J. Schoenmakers, B. W. J. Pirok and M. R. van Bommel, *J Chromatogr A*, 2023, **1688**, 463723.

53 S. Torres, R. Brown, T. Zelesky, G. Scrivens, R. Szucs, J. M. Hawkins and M. R. Taylor, *J Pharm Biomed Anal*, 2016, **131**, 71–79.

54 M. Karady, O. Novák, A. Horna, M. Strnad and K. Doležal, *Electroanalysis*, 2015, **27**, 406–414.

55 M. Szultka-Młyńska, S. Bajkacz, I. Baranowska and B. Buszewski, *Talanta*, 2018, **176**, 262–276.

56 M. Szultka-Młyńska, S. Bajkacz, M. Kaca, I. Baranowska and B. Buszewski, *Journal of Chromatography B*, 2018, **1093–1094**, 100–112.

57 M. Szultka-Mlynska and B. Buszewski, *J Pharm Biomed Anal*, 2019, **176**, 112799.

58 T. F. Mekonnen, U. Panne and M. Koch, *Anal Bioanal Chem*, 2018, **410**, 2607–2617.

59 C. K. Mak, C. A. Wehe, M. Sperling and U. Karst, *J Chromatogr A*, 2015, **1419**, 81–88.

60 Navarro Suarez, Thein, Kallinich and Rohn, *Molecules*, 2019, **24**, 2369.

61 Z.-X. Yue, Y.-X. Gu, T.-C. Yan, F.-M. Liu, J. Cao and L.-H. Ye, *Food Chem*, 2022, **380**, 132202.

62 L. Knoche, J. Lisec and M. Koch, *Rapid Communications in Mass Spectrometry*, , DOI:10.1002/rcm.9349.

63 Q. Zheng, Z. Wang, A. Xiong, Y. Hu, Y. Su, K. Zhao and Y. Yu, *Food Chem*, 2021, **355**, 129596.

64 R. C. Tonleu Temgoua, U. Bussy, D. Alvarez-Dorta, N. Galland, E. Njanja, J. Hémez, C. Thobie-Gautier, I. K. Tonlé and M. Boujtita, *Environ Sci Process Impacts*, 2021, **23**, 1600–1611.

65 T. F. Mekonnen, U. Panne and M. Koch, *Anal Bioanal Chem*, 2017, **409**, 3359–3368.

66 U. Bussy, Y.-W. Chung-Davidson, K. Li and W. Li, *Environ Sci Technol*, 2015, **49**, 4450–4457.

67 T. Wigger, A. Seidel and U. Karst, *Chemosphere*, 2017, **176**, 202–211.

68 B. Seiwert, N. Golan-Rozen, C. Weidauer, C. Riemenschneider, B. Chefetz, Y. Hadar and T. Reemtsma, *Environ Sci Technol*, 2015, **49**, 12342–12350.
